# Analysis of novel *RUNX2* mutations in Chinese patients with cleidocranial dysplasia

**DOI:** 10.1371/journal.pone.0181653

**Published:** 2017-07-24

**Authors:** Xianli Zhang, Yang Liu, Xiaozhe Wang, Xiangyu Sun, Chenying Zhang, Shuguo Zheng

**Affiliations:** Department of Preventive Dentistry, Peking University School and Hospital of Stomatology, National Engineering Laboratory for Digital and Material Technology of Stomatology, Beijing Key Laboratory of Digital Stomatology, Beijing, PR China; NIDCR/NIH, UNITED STATES

## Abstract

Cleidocranial dysplasia (CCD) is an autosomal dominant inheritable skeletal disorder characterized by cranial dysplasia, clavicle hypoplasia and dental abnormalities. This disease is mainly caused by heterozygous mutations in *RUNX2*, a gene that encodes an osteoblast-specific transcription factor. In the present study, mutational analyses of *RUNX2* gene were performed on four unrelated Chinese patients with CCD. Four different *RUNX2* mutations were detected in these patients, including one nonsense mutation (c.199C>T p.Q67X) and three missense mutations (c.338T>G p.L113R, c.557G>C p.R186T and c.673C>T p.R225W). Among them, two mutations (c.199C>T p.Q67X and c.557G>C p.R186T) were novel and the other two had been reported in previous literatures. Except for Q67X mutation located in the Q/A domain, other three mutations were clustered within the highly conserved Runt domain. Green fluorescent protein (GFP) and RUNX2 fusion protein analyses *in vitro* showed that nuclear accumulation of RUNX2 protein was disturbed by Q67X mutation, while the other two mutations (c.338T>G p.L113R and c.557G>C p.R186T) had no effects on the subcellular distribution of RUNX2. Luciferase reporter assay demonstrated that all the three novel *RUNX2* mutations significantly reduced the transactivation activity of RUNX2 on *osteocalcin* promoter. Our findings enrich the evidence of molecular genetics that the mutations of *RUNX2* gene are responsible for CCD.

## Introduction

Cleidocranial dysplasia (CCD, OMIM 119600) is an autosomal-dominantly inherited skeletal disorder, which is characterized by persistently open or delayed closure of cranial sutures, hypoplastic or aplastic clavicles, and many dental abnormalities, including delayed eruption of secondary teeth accompanied by retained primary teeth, and multiple supernumerary teeth [1]. In addition, hand abnormalities could also be observed in some patients, such as brachydactyly, tapering fingers, and short, broad thumbs [1]. Individuals with CCD are shorter than their unaffected sibs and are more likely to have other skeletal/orthopedic problems such as pes planus, genu valgum, and scoliosis [1, 2]. The spectrum of phenotypes is dramatically variable even within families, ranging from only mildly affected patients with dental abnormalities to severely affected ones with severe defects in skeletal system [3, 4].

Runt-related transcription factor 2 (*RUNX2*, also known as *CBFA1*) has been identified as the causing gene for CCD [1, 5]. The human *RUNX2* gene is mapped to chromosome 6p21, consisting of eight coding exons [6]. It is revealed that the osteoblast-specific transcription factor RUNX2 promotes the differentiation of mesenchymal cells into osteoblasts in the cartilage anlagen [7]. Homozygous *Runx2* knockout mice (*RUNX2*^**-/-**^) will die of respiratory failure soon after birth and shows failure in both intramembranous and endochondral ossification, whilst heterozygous *Runx2* knockout mice (*RUNX2*^**+/-**^) displays similar phenotype with CCD patients in hypoplastic clavicles and defective skull formation [8].

The RUNX2 protein contains an N-terminal stretch of consecutive ployglutamine and polyalanime repeats (the Q/A domain), a Runt domain and a C-terminal proline/serine/threomine-rich (PST) activation domain [9]. The Runt domain is a characteristic and highly conserved motif homologous to pair-rule *runt* gene in *Drosophila* segmentation and can be detected in all the three members of the RUNX family [6]. The Runt domain of *RUNX2* is mainly responsible for DNA-binding to a specific motif and heterodimerization with core-binding factor β (CBFβ), a non-DNA-binding subunit [10, 11]. The resulting complex binds to cis-acting elements of its targets and regulates the expression of skeletal formation-related genes, such as *osteocalcin* and *osteopontin* [7]. The nuclear localization signal (NLS) is located on the carboxyl-terminal border of the Runt domain, and regulates the accumulation of the RUNX2 protein into nuclei [12, 13]. The PST domain is necessary for RUNX2-mediated transcriptional regulation and is involved in functional interactions with a number of other transcription factors, co-activators and co-repressors [14]. It is reported that the Q/A domain is also involved in the regulation of the transactivation activity of RUNX2 target genes [15].

Up to the present, at least 177 heterozygous mutations in the *RUNX2* gene have been identified in CCD patients, majority of which were missense, nonsense and frameshift mutations (S1 Table) [16]. Chromosomal translocations, splicing mutation, and intragenic deletions / duplications were also found and they were scattered throughout the entire *RUNX2* gene [17].

Previously, our research group had reported six unrelated Chinese patients with classic CCD phenotype and identified five mutations in the coding region of the *RUNX2* gene [4, 18]. The present study aims to investigate potentially novel mutations of the *RUNX2* gene and their impacts on the functions of the RUNX2 protein in patients with CCD, and to explore the genotype-phenotype correlation of this syndrome.

## Materials and methods

### Patients

Four unrelated Chinese individuals were referred to Peking University School and Hospital of Stomatology with main complaint of retention of deciduous teeth. Clinical and radiographic examinations were performed and they were diagnosed as CCD according to the criteria of clinical diagnosis [1]. From March 2014 to May 2016, the CCD patients and their family members were recruited to participate in this study with written consent informed from participants or guardians of the minors. This study was ethically approved by the Ethical Committee of Peking University School and Hospital of Stomatology (issue number: PKUSSIRB-2012004). The individuals in this manuscript had given us their written informed consents to publish these case details.

### Mutation analysis

Genomic DNA was extracted from peripheral blood samples of the participants using TIANamp Blood DNA mini kit (TIANGEN, Beijing, PR China) according to the manufacturer’s instruction. The exons (0–7) and exon-intron boundaries of the *RUNX2* gene were amplified by polymerase chain reaction (PCR). The primers used for both genomic PCR amplification and Sanger sequencing were according to previous literature [12]. In brief, the PCR reactions was carried out using the following program: an initial denaturation at 94°C for 5 min, then 35 cycles of 94°C for 20 sec, 62°C for 30 sec, and 72°C for 1 min followed by a final extension at 72°C for 7 min. Each reaction of PCR contained 50 ng genomic DNA. DNA Polymerase was purchased from Takara Technologies. Dimethyl sulphoxide (Sigma-Aldrich, St. Louis, MO, USA) was added to the Exon1 amplifying system at a final concentration of 10%. After purification, the products of amplification were bi-directionally sequenced in ABI 3730 XL automatic sequencer (Applied Biosystems, Foster city, CA, USA). DNA sequences were analyzed using all the databases of NCBI and the BLASTN (BLAST nucleotide) program (http://blast.ncbi.nlm.nih.gov/). The exons are numbered according to GenBank entries AF001443–AF001450. Each mutation was confirmed in at least two independent experiments by nucleotide sequencing.

### Damaging effects prediction and conservation analysis

Three online programs, including PolyPhen-2 (http://genetics.bwh.harvard.edu/pph2/), SIFT (http://sift.jcvi.org) and Mutation Taster (http://www.mutationtaster.org), were used to predict the effects of the three missense mutations on the function of the RUNX2 protein. The conservation characteristics of the affected amino acids among nine species were analyzed using the library of HomoloGene database (http://www.ncbi.nlm.nih.gov/homologene). Furthermore, the minor allele frequency of each mutation was analyzed using ExAC database (http://exac.broadinstitute.org).

### Mutagenesis

Wild-type pEGFPN1-RUNX2 plasmid and pCMV5-RUNX2 plasmid (a generous gift of Dr. Renny T. Franceschi, School of Dentistry, University of Michigan) were used as previously described [4, 18]. Amino acid substitution mutations were introduced to *RUNX2* using QuikChange Lightning Site-Directed Mutagenesis Kit (Agilent Technologies, Cedar Creek, TX, USA) according to the manufacturer’s protocol. All plasmids were fully sequenced in order to confirm the target mutations and exclude any additional mutations.

### Cell culture

Mouse preosteoblast MC3T3-E1 cells (a generous gift of Dr. Hailan Feng, Department of Prosthodontics, Peking University School and Hospital of Stomatology) were cultured in α-minimum essential medium (Life Technologies Corporation, Grand island, NY, USA) supplemented with 10% fetal bovine serum (Life Technologies Corporation) and 100U/ml penicillin-streptomycin (Life Technologies Corporation) at 37°C in the humidified air containing 5% CO_2_.

### Subcellular localization assay

MC3T3-E1 cells were seeded onto microscopic cover glass with the cell density of 1x10^**5**^/well in 6-well plate. After overnight incubation, the cells were transfected with 2.5 μg GFP control plasmid or wild-type/mutant-type pGFP-RUNX2 constructs using 3.75 μL Lipofectamine 3000 transfection reagent (Life Technologies Corporation) according to the manufacturer’s instruction. At 48 h post-transfection, cells were fixed with 4% paraformaldehyde in PBS for 10 min at room temperature. The coverslips were mounted with DAPI-containing fluoroshield mounting (Sigma-Aldrich, St. Louis, MO, USA). Cells were visualized and captured using Zeiss LSM 5 EXCITER confocal microscope (Carl Zeiss, Jena, TH, Germany).

### *Osteocalcin* promoter luciferase assay

On the day before transfection, MC3T3-E1 cells were seeded onto 24-well plate with the cell density of 4×10^4^ cells per well. When MC3T3-E1 cells reached 60–70% confluence, transient transfection was carried out using Lipofectamine 3000 (Life Technologies Corporation) according to the protocol provided by manufacturer. Each transfection was performed with 200ng wild-type/mutant-type pCMV5-Runx2 constructs which acted as effector plasmid, 200ng p6OSE2-luc reporter gene construct (provided by Dr. Gerard Karsenty, Department of Genetics and Development, Columbia University, NY, USA) and 0.4ng pRL-TK Renilla luciferase vector (provided by Dr. Tiejun Li, Department of Pathology, Peking University School and Hospital of Stomatology, Beijing, PR China) as the internal control to normalize the transfection efficiency. At 24 h post-transfection, cells were collected and lysed, then luciferase activities were measured using Dual-Luciferase Reporter Assay kit (Promega, Madison, WI, USA). All transfection experiments described here were performed in triplicate and repeated for at least three times.

### Statistical analysis

Data were presented in form of means ± SD. Statistical analyses were performed using two-tailed Student’s *t*-test. *p* value < 0.05 was considered as statistical significance.

## Results

### Clinical findings

Four unrelated Chinese patients were included in this study (Table 1). Among them, 2 families were familial cases, and the other two were sporadic ones. All the patients showed typical features of CCD, such as hypoplastic clavicles, patent fontanelles and multiple dental anomalies, including retained deciduous teeth, delayed eruption of permanent teeth. Notably, supernumerary teeth were existed in all the patients, while the number of supernumerary teeth showed moderate variation between patients. Furthermore, stature was found to be significantly short in male patients with CCD compared to the average level of the Chinese population.

**Table 1:** Molecular and clinical features of the four CCD patients in this study.

Patient ID	Family history	Sex^a^	Age^b^ (years)	Stature (cm)	Patent fontanelles	Hypoplastic clavicles^c^	Number of supernumerary teeth	Deciduous teeth retention	Delayed eruption of permanent teeth	Mutation			
										Nucleotide^d^	Codon	Type	Location
1	+	F	14	163	+	+	12	+	+	c.199C>T	Q67X	Nonsense	Q/A
2	+	M	16	167	+	+	7	+	+	c.338T>G	L113R	Missense	Runt
3	-	F	42	156	+	+	6	+	+	c.557G>C	R186T	Missense	Runt
4	-	M	12	145	+	++	4	+	+	c.673C>T	R225W	Missense	Runt

^a^ F, female; M, male.

^b^ Age referred to the year when their body height was measured.

^c^ +, Hypoplastic clavicles; ++, aplastic clavicles.

^d^ Numbering is according to the NM_001024630.3 transcript variant.

**Fig 1:**
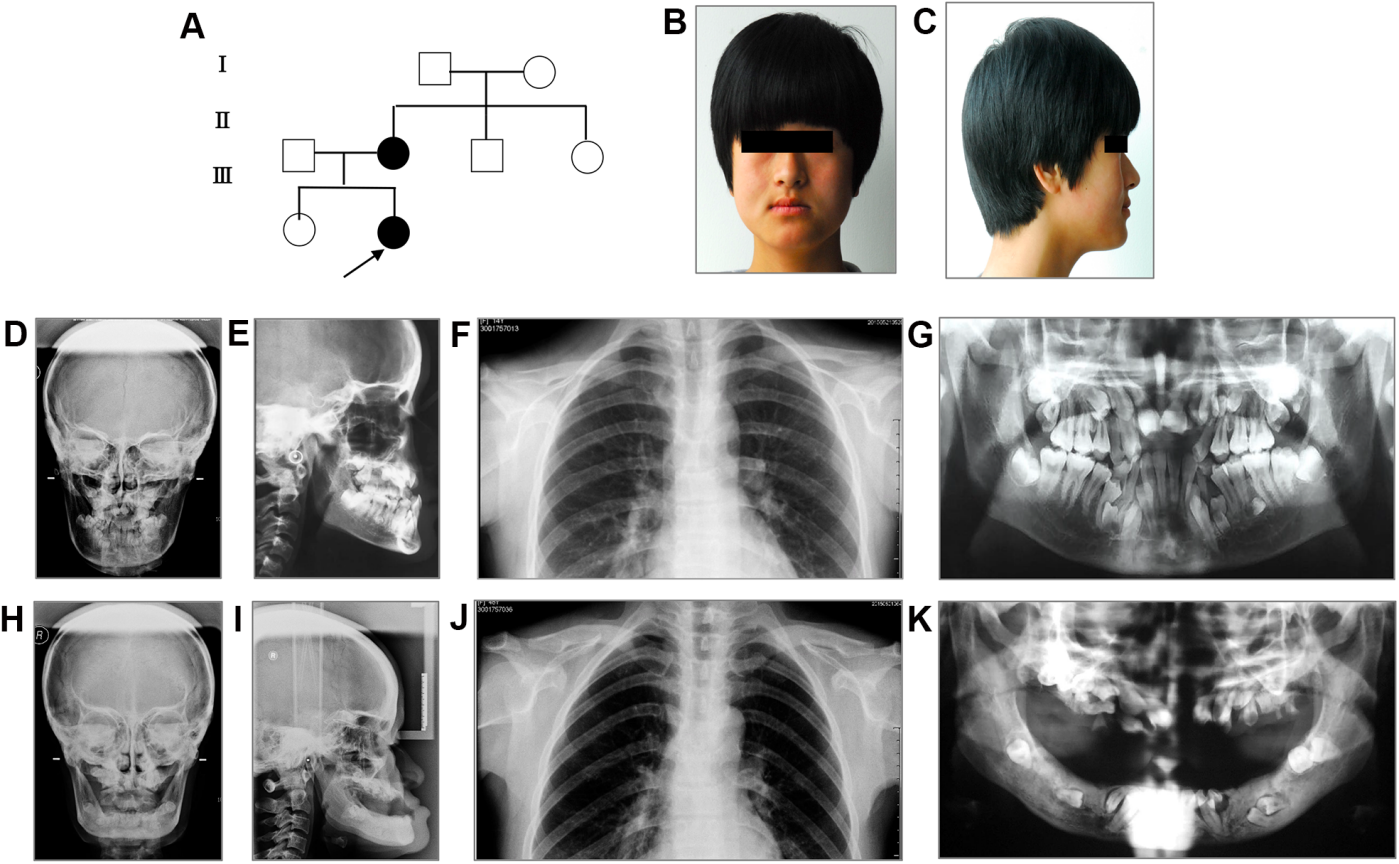
Clinical and radiological findings in the family members of Patient 1. (A) Pedigree of Patient 1. The arrow indicates the proband. (B, C) Clinical facial photographs of the proband. (D-G) Radiographs of the proband. Anteroposterior (D), lateral (E), Chest (F), and panoramic (G) radiograph of the proband. (H-K) Radiographs of the proband's mother. Anteroposterior (H), lateral (I), chest (J), and panoramic (K) radiograph of the proband's mother.

Patient 1 was a familial case with two family members affected in this family (Fig 1A). The proband, a 14-year-old girl, had classic craniofacial features like concave facial type and mid-face dysplasia (Fig 1B and 1C). This patient also exhibited delayed closure of sagittal suture (Fig 1D and 1E), bilateral hypoplastic clavicles (Fig 1F), and multiple dental abnormalities such as crossbite, retained deciduous teeth, delayed eruption of permanent teeth and supernumerary teeth (Fig 1G). The phenotype of the proband's mother was similar to the proband (Fig 1H–1J). As a middle-aged person, it was striking that a complete denture was already present in her mandible. Several deciduous teeth were still retained and many permanent teeth including supernumerary teeth were embedded in the mandible bones (Fig 1K).

**Fig 2:**
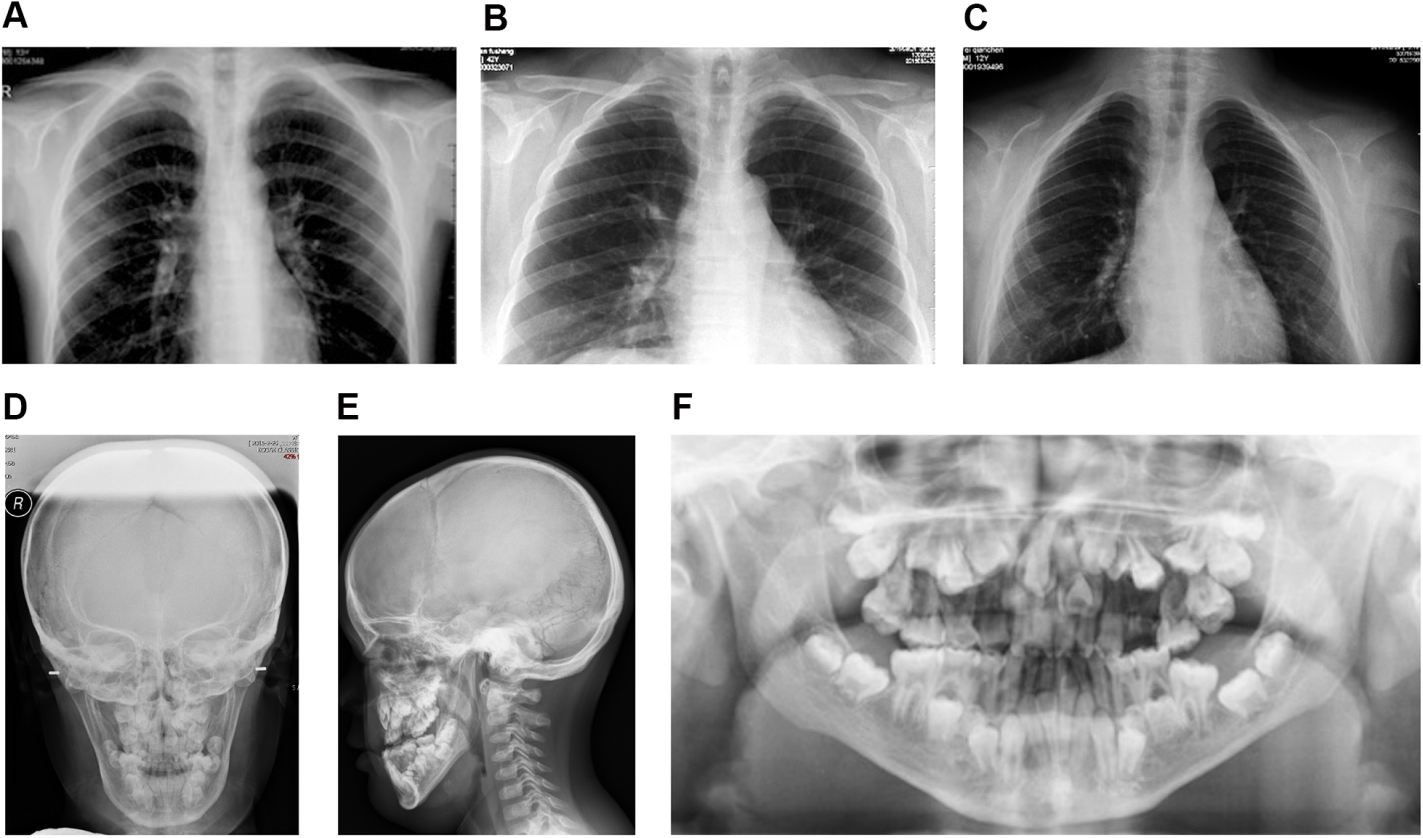
Radiographic manifestations of Patient 2, Patient 3 and Patient 4. (A-C) Chest radiographs of Patient 2 (A), Patient 3 (B), and patient 4 (C). (D-F) Clinical radiographs of Patient 4. Anteroposterior (D), lateral (E), and panoramic radiographs (F) of Patient 4.

The overall phenotype of other three CCD patients was similar to patient 1, although the severity of hypoplastic clavicles had an obvious variation among them (Fig 2A–2C). Bilateral clavicles of the patient 4 were aplasic (Fig 2C), while for patient 2 (Fig 2A) and patient 3 (Fig 2B), the bilateral clavicles were hypoplastic. Besides, open fontanelles, delayed closure of sutures, multiple Wormian bones in the lambdoid and sagittal sutures were also observed in patient 4 (Fig 2D and 2E). Multiple dental abnormalities, including retained deciduous teeth, delayed eruption of permanent teeth and supernumerary teeth could be observed in patient 4 (Fig 2F).

### Mutation analysis

**Fig 3:**
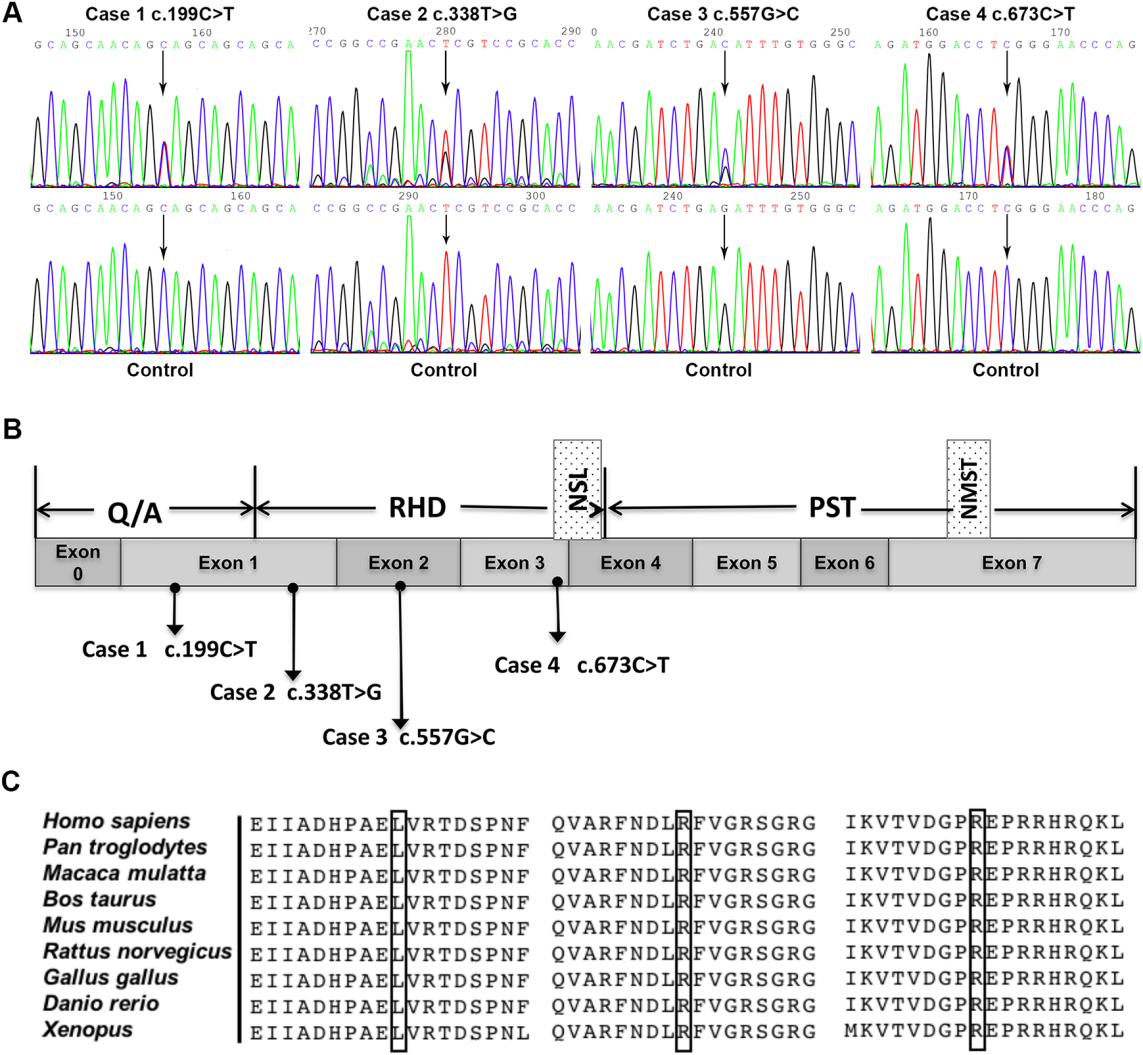
Mutation analysis of *RUNX2* gene for the four CCD patients. (A) Identification of four different heterozygous mutations in *RUNX2* gene from four CCD patients. For each mutation, the mutated sequence (upper panel) and control sequence (lower panel) were shown respectively. (B) Schematic presentation of the *RUNX2* gene structure and annotated mutations identified in our cases. The bar depicts the structure of human RUNX2 protein. Q/A, Glutamine-alanine repeat domain; RHD, DNA-binding Runt homology domain; NLS, nuclear localization signal; PST, proline/serine/threonine rich region; NMST, unclear matrix targeting sequence. (C) Partial amino acid sequences alignment in RUNX2 protein among nine species. The positions of the mutated amino acid in our study are indicated using black boxes.

Sequencing analysis was performed in the coding region of *RUNX2* gene. Four different heterozygous mutations were detected in the four CCD pedigrees, including one nonsense mutation and three missense mutations (Table 1 and Fig 3A). All the detected mutations were clustered in the highly conserved Runt domain except for the nonsense mutation, which was located in the Q/A domain (Fig 3B). Minor allele frequency of the three novel mutations was also analyzed in ExAC database to verify our finding. Our analysis showed that neither of the three variants were found in ExAC database (Data not shown).

### Nonsense mutation

In patient 1, a heterozygous c.199C>T nonsense mutation was detected in exon 1 of the *RUNX2* gene (Fig 3A). This novel mutation introduced a new stop codon at amino acid position 67, leading to a premature protein truncated at the polyglutamine stretch of the Q/A domain. Consistent with its phenotype, this mutation was also detected in the proband’s mother who also showed typical CCD features, but not in other family members.

### Missense mutations

In our patient cohort, three missense mutations were detected in the three unrelated families. One mutation (c.557G>C, p.R186T) was novel, and the other two (c.338T>G, p.L113R and c.673C>T, p.R225W) had been described previously [12]. Partial amino acid sequences alignment in the RUNX2 protein among nine species showed that the affected amino acids, namely L113, R186 and R225, were found to have a high level of evolutionary conservation among species (Fig 3C). Prediction of damaging effects of the three missense mutations were performed using three different *in silico* programs, including PolyPhen-2, SIFT and Mutation Taster. All the predicted results showed that these missense mutations were deleterious and severely damaged the function of RUNX2 (Table 2).

**Table 2:** Predicted damaging effects of missense mutations in *RUNX2* gene.

Gene symbol	Amino acid change	Polyphen-2 (Score)	SIFT (Score)	Results of Mutation taster
*RUNX2*	p.L113R	Probably damaging (0.960)	Deleterious (0.00)	“Prediction disease causing”
*RUNX2*	p.R186T	Probably damaging (1.000)	Deleterious (0.00)	“Prediction disease causing”
*RUNX2*	p.R225W	Probably damaging (1.000)	Deleterious (0.00)	“Prediction disease causing”

In case 2, a T to G substitution at nucleotide 338 was identified in exon 1 of *RUNX2* causing leucine 113 convert to arginine (Fig 3A), which had been reported previously [12]. However, only one mutation (L113R) was detected in the present study which is different from above study, in which two different missense mutations (L113R and C123R) were detected in exon 1 [12]. Consistent to the clinical finding, the L113R mutation was also detected in the *RUNX2* gene of patient 2’s father (data not shown).

Patient 3 was a sporadic case in which a heterozygous G to C transition at nucleotide position 557 was identified in exon 2 of *RUNX2* (Fig 3A). This mutation resulted in arginine 186 convert to threonine. Patient 4 was also a sporadic case. A heterozygous mutation 673C to T in exon 3 of *RUNX2* gene was detected in this patient (Fig 3A). This mutation resulted in arginine to tryptophan transition at amino acid position 225 in the Runt domain.

### Subcellular localization of the Runx2 mutants

As a transcription factor, in physiological conditions RUNX2 was located in the nucleus under the guidance of NLS, which was at the C-terminal border of the Runt domain. Therefore, it was hypothesized that mutations affecting NLS could interfere with the subcellular distribution of RUNX2.

**Fig 4:**
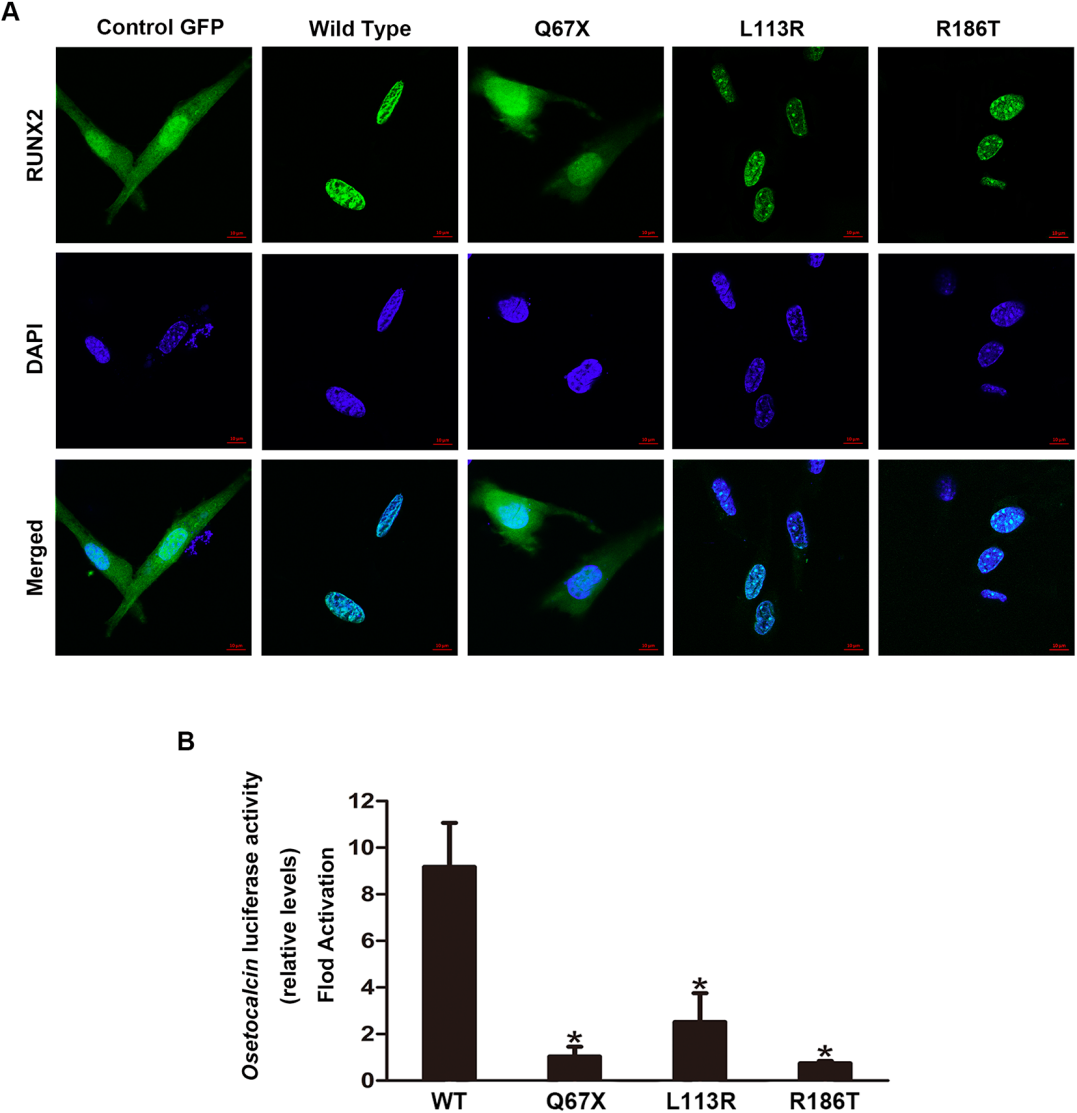
Functional analyses of *RUNX2* mutations on protein subcellular localization and transactivation of down-stream target gene. (A) Subcellular localization of wild-type and mutant RUNX2-GFP fusion proteins in MC3T3-E1 cells. Confocal micrographs showed the intracellular distribution of control GFP, wild-type RUNX2-GFP, Q67X, L113R, and R186T mutant proteins. (B) Transactivation activity of mutant RUNX2 proteins on *osteocalcin* promoter. MC3T3-E1 cells were co-transfected with a control pCMV5 vector or a vector that expressed wild-type or mutant RUNX2 protein, together with *osteocalcin* reporter plasmid. *Renilla* luciferase reporter was used as the internal control. Bars represent firefly/Renilla luciferase ratios for different constructs. *p < 0.05 (compared with wild-type control).

To test this hypothesis, a RUNX2 reporter with green fluorescent protein (GFP) tag at the C-terminus of pEGFP-N1 vector was constructed. All the detected novel mutations were introduced into wild-type pEGFPN1-RUNX2 construct by mutagenesis, then the constructs were transiently transfected into MC3TC-E1 cells. In contrast to GFP control which was located in both the cytoplasm and the nucleus (Fig 4A), wild-type RUNX2-GFP fusion protein was predominantly distributed in the nucleus (Fig 4A). The Q67X mutant resulted in a truncated protein in which NLS was absent. As expected, Q67X mutant was equi-distributed in both the nucleus and the cytoplasm (Fig 4A) which was quite different from the wild-type RUNX2. On the contrary, the other two mutants (L113R, R186T) showed a similar subcellular distribution with the wild-type RUNX2 as a result of the mutations had no effects on NLS (Fig 4A).

### Transactivation abilities of the *RUNX2* mutants on target gene

To investigate the effects of the *RUNX2* mutations on the transactivation ability of the RUNX2 protein on its target gene, the co-transfection assay was performed. In this experiment, the plasmid with a RUNX2-responsive *osteocalcin* promoter linked to the *luc* gene (p6OSE2-luc) was used as the reporter, which has been well-characterized as an osteoblast-specific target of RUNX2 [19]. The luciferase reporter assay showed that all the mutants significantly reduced the transactivation ability of RUNX2 on *osteocalcin*, one of its downstream target (Fig 4B).

## Discussion

In the present study, four different heterozygous mutations in the *RUNX2* gene were identified in four unrelated Chinese CCD families, including one nonsense mutation and three missense mutations. Among these mutations, two were novel, and the other two had been reported previously [12]. All the detected mutations were clustered in the Runt domain except for the nonsense mutation, which was located in the Q/A domain. This is consistent with previous literatures that most of the missense mutations in CCD patients were located within the DNA-binding region of the Runt domain [16, 20–22]. This indicated that the Runt domain, which had highly conserved sequence, was very susceptible to amino acid substitution [18, 23]. Meanwhile, missense mutations were rarely detected in the Q/A and PST domains [16, 24].

In our patient cohort, though all the patients displayed classic CCD phenotypes with hypoplastic clavicles, delayed closure of the fontanelle and dental abnormalities, there is a moderate range of clinical expressivity of phenotype. In this study, short stature was found only in male patients, but we cannot relate it to altered Runt domain as suggested elsewhere [23]. This finding was agreed with previous reports published by our group and others [4, 25]. Supernumerary teeth are usually considered to be a diagnostic feature of CCD. However, the number of supernumerary teeth had a clear variation among patients in the present study. From our previous clinical observations, sometimes the number of supernumerary teeth can be age-dependent in CCD patients, and exact quantification is only possible in adult patients considering all surgical interventions. Therefore, we could not definitely correlate the quantity of supernumerary teeth or body height with altered Runt domain as reported by Yoshida et al [23].

The *RUNX2* gene is one of the three mammalian genes encoding the α subunit of the heterodimeric transcription factor PEBP2/CBF [26]. PEBP2/CBF is made of two structurally unrelated subunits, α and β [26]. The other two α encoding genes are RUNX1 [15,16] and RUNX3 [6,17,18]. On the other hand, only one gene is known to encode the mammalian β subunit, termed PEBP2β/CBFβ [14,24], which has been demonstrated to be essential for both hematopoiesis [10, 27] and osteogenesis [27–29]. The α subunit is characterized by a highly conserved 128-amino acid region termed the Runt domain, which is responsible for DNA- binding and heterodimerization with the β subunit [26]. Functional studies performed on RUNX1 verified the important role of Runt domain. One study found that R135 and R174 in the Runt domain of RUNX1, which corresponded to R186 and R225 in the Runt domain of RUNX2, directly interacted with DNA but not CBFβ [28]. And the functional assays found that DNA-binding abilities in R135G, R135A and R174W mutants of RUNX1 were diminished, while the capability of heterodimerization with CBFβ still remained [29]. Therefore, the R186T and R225W mutants found in RUNX2 were predicted to abolish the DNA-binding ability of the transcription factor, but not the capability of heterodimerization with CBFβ, though we did not test this functionally. The L113 amino acid residue in the Runt domain of RUNX2, which was homologous to L62 in the Runt domain of RUNX1, was involved in stabilizing the domain structure [29]. Furthermore, functional analyses indicated that this mutation in this leucine was defective for both DNA-binding ability and heterodimerization with CBFβ [29]. As the amino acid sequences of the Runt domain exhibited more than 90% identity among the RUNX family, it is suggested that the similar mechanisms in governing the functions of DNA-binding ability and heterodimierzation among the entire family of the RUNX family [28–30].

The NLS of the RUNX2 protein was first delineated as a stretch of nine amino acids (PRRHRQKLD) on the carboxyl-terminal border of the Runt domain in the RUNX2 protein [31]. As the NLS was responsible for the nuclear localization of the RUNX2 protein, we hypothesized that mutations affecting the NLS region may interfered with the subcellular distribution of RUNX2. To confirm this, the RUNX2-GFP fusion protein reporter was constructed and transient transfection assay was performed. Our GFP reporter assay showed that the Q67X mutation interfered with subcellular localization of RUNX2. This is expectable since the Q67X mutation produced a premature protein truncated at the Q/A domain with only 67 amino acid residues, leading to the absence of other key domains, including the Runt domain, the NLS and the PST domain. This is consistent with our previous study that 172fs and 214fs mutations caused the mutant RUNX2 proteins unable to accumulate in the nucleus as a result of the lack of NLS (4). Other studies also found that *RUNX2* mutations could cause NLS deletion or amino acid changes which would interfere with the subcellular distribution of RUNX2 (12, 22, 25). In contrast, L113R, R186T mutants were missense mutations located in the Runt domain and outside of NLS. Consistently, our GFP reporter assay showed these mutations had no effects on subcellular distribution of RUNX2, which was also reported in previous studies [4].

In this study, our transactivation assay revealed that both direct substitution of conserved amino acid residues (L113R, R186T) and truncated protein (Q67X) could result in a significant reduction of the transactivation ability of RUNX2 on *osteocalcin*. These findings are consistent with previous studies by our research group and other researchers [18, 23, 32, 33]. These results are predictable regarding the crucial role of the Runt domain on RUNX2 functions. Q67X mutation would result in the deletion of the Runt domain, whilst the other two missense mutations (L113R, R186T) were happened in the Runt domain. It is widely known that the Runt domain was essential for RUNX2 to form heterodimerization with CBFβ and bind to the promoters of its downstream target genes [10, 11]. Therefore, *RUNX2* mutations could impair the Runt domain in both the DNA-binding ability and subsequent transactivation [23, 33]. In the present study, all the four CCD patients showed typical manifestations of CCD clinically with similar phenotypes. Thus, for the three patients with missense mutations in the Runt domain, impaired transactivation activities of RUNX2 might be one potential reason of why this kind of patients showed typical manifestations of CCD though their subcellular distribution of RUNX2 was not affected.

## Conclusion

In conclusion, four CCD patients were included in this study and four different heterozygous mutations in the *RUNX2* gene were identified. The c.199C>T p.Q67X mutation not only interfered with the subcellular distribution of RUNX2, but also reduced the transactivation activity of RUNX2 on its downstream target genes. Other two missense mutations (c.338T>G p.L113R and c.557G>C p.R186T) located in the Runt domain severely impaired the transactivation activity of RUNX2 on its downstream target genes, though they had no effects on the subcellular distribution. From these findings, it is expected that the impaired transactivation activity of RUNX2 on its downstream target genes would be responsible for the cause of haploinsufficiency in these cases with CCD.

## Supporting information

S1 Table Known mutations in *RUNX2* gene in cleidocranial dysplasia. (DOCX)
