# Supplementary material for: Analysis of novel RUNX2 mutations in Chinese patients with cleidocranial dysplasia
Source: PLoS One. 2017 Jul 24;12(7):e0181653. doi: 10.1371/journal.pone.0181653 (PMC5524338; doi:10.1371/journal.pone.0181653)
Supplement: S1 Table — (DOCX) [file pone.0181653.s001.docx]

**S1 Table. Known Mutations in *RUNX2* gene in cleidocranial dysplasia**

| Exon number | Mutation types | | | | |
| --- | --- | --- | --- | --- | --- |
|  | Nonsense | Missense | Small deletions | Small insertions |  |
| Exon 0 (1-20 aa) |  |  |  |  |  |
| Exon 1 (20-141 aa) | c.148C>T p.Q50X | c.158A>T p.Q53L | c.178delC | c.90_91insC |  |
|  | c.169C>T p.Q57X | c.338T>G p.L113R | c.221_266del47 | c.92_93insCGGT |  |
|  | c.190C>T p.Q64X | c.353G>A p.S118N | c.230_276del | c.134_135insGTCC |  |
|  | c.193C>T p.Q65X | c.354C>A p.S118R | c.274delC | c.181_189dupGCGGCGGCT |  |
|  | c.196C>T p.Q66X | c.354C>G p.S118R | c.282delG | c.186ins(16) |  |
|  | c.273T>A p.L93X | c.362T>G p.F121C | c.329delC | c.207-208ins13 |  |
|  | c.334G>T p.E112X | c.380C>T p.P127L | c.220del172 | c.222ins(30) |  |
|  | c.383C>A p.S128X | c.386A>G p.H129R |  | c.276_277ins17 |  |
|  | c.389G>A p.W130X | c.388T>A p.W130R |  | c.278insT |  |
|  | c.396C>A p.C132X | c.391C>A p.R131S |  | c.295insA |  |
|  |  | c.391C>G p.L131G |  | c.382_383insT |  |
|  |  | c.391C>T p.R131C |  | c.398_399ins(15) |  |
|  |  | c.407T>C p.L136P |  | c.411_412insG |  |
| Exon 2 (142-194 aa) | c.436G>T p.G146X | c.436G>A p.G146R | c.453delT | c.469insA |  |
|  | c.484G>T p.E162X | c.467T>A p.V156D | c.476delG | c.470dupT |  |
|  | c.577C>T p.R193X | c.467T>G p.V156G | c.481_482delGA | c.522insA |  |
|  |  | c.475G>C p.G159R | c.495delT |  |  |
|  |  | c.509G>A p.R169Q | c.514delC |  |  |
|  |  | c.509G>C p.R169P | c.532delC |  |  |
|  |  | c.524T>A p.M175K | c.540_549del(10) |  |  |
|  |  | c.524T>G p.M175R | c.549delT |  |  |
|  |  | c.523A>G p.M175V | c.553_554delCT |  |  |
|  |  | c.560T>C p.F187S |  |  |  |
|  |  | c.569G>A p.R190Q |  |  |  |
|  |  | c.569G>C p.R190P |  |  |  |
|  |  | c.568C>T p.R190W |  |  |  |
|  |  | c.572G>A p.S191N |  |  |  |
|  |  | c.574G>A p.G192R |  |  |  |
|  |  | c.578G>A p.R193Q |  |  |  |
| Exon 3 (194-229 aa) | c.625C>T p.Q209X | c.590T>C p.F197S | c.592delA | c.625_624insCC |  |
|  | c.652A>T p.K218X | c.597G>C p.L199F | c.604delA | c.631_633GCT |  |
|  |  | c.599C>T p.T200I | c.636delC |  |  |
|  |  | c.598A>G p.T200A | c.644delG |  |  |
|  |  | c.602T>A p.I201K |  |  |  |
|  |  | c.614C>G p.T205R |  |  |  |
|  |  | c.627A>T p.Q209H |  |  |  |
|  |  | c.626A>G p.Q209R |  |  |  |
|  |  | c.631G>C p.A211P |  |  |  |
|  |  | c.654A>T p.K218N |  |  |  |
|  |  | c.652A>C p.K218Q |  |  |  |
|  |  | c.652A>G p.K218E |  |  |  |
|  |  | c.657C>T p.T220I |  |  |  |
|  |  | c.662T>G p.V221G |  |  |  |
|  |  | c.667G>A p.G223R |  |  |  |
|  |  | c.670C>T p.P224S |  |  |  |
|  |  | c.673C>T p.R225W |  |  |  |
|  |  | c.674G>A p.R225Q |  |  |  |
|  |  | c.674G>T p.R225L |  |  |  |
|  |  | c.682A>G p.R228G |  |  |  |
| Exon 4 (229-287 aa) | c.694C>T p.Q232X | c.859G>A p.D287N | c.716delC |  |  |
|  | c.838C>T p.Q280X |  | c.718_721del |  |  |
|  |  |  | c.722delT |  |  |
|  |  |  | c.821delC |  |  |
|  |  |  | c.821_822delCA |  |  |
|  |  |  | c.824delG |  |  |
|  |  |  | c.831delT |  |  |
| Exon 5 (287-341 aa) | c.868C>T p.Q290X |  | c.873_874delCA | c.887insC |  |
|  | c.891G>A p.W297X |  | c.879_885del |  |  |
|  |  |  | c.884delC |  |  |
|  |  |  | c.887delC |  |  |
|  |  |  | c.915delG |  |  |
|  |  |  | c.977delG |  |  |
| Exon 6 ( 341-363aa) | c.1055G>A p.W352X |  |  |  |  |
| Exon 7 (363-521 aa) | c.1096G>T p.E366X | c.1259C>Ap.T420N | c.1111_1129del19 | c.1111dupT |  |
|  | c.1171C>T p.R391X | c.1259C>T p.T420I | c.1119delC | c.1116_1119insC |  |
|  | c.1182T>A p.Y394X | c.1565G>C p.X522S | c.1121delG | c.1123_1124insA |  |
|  | c.1254C>A p.Y418X |  | c.1131delA | c.1127_1128insT |  |
|  |  |  | c.1153_1154del | c.1204-1205insACCC |  |
|  |  |  | c.1157delG | c.1205_1206insC |  |
|  |  |  | c.1169delC | c.1215_1216insC |  |
|  |  |  | c.1205delC | c.1228insC |  |
|  |  |  | c.1250_1251delAC | c.1250insA |  |
|  |  |  | c.1335_1336delCT | c.1271_1272ins(20) |  |
|  |  |  | c.1550_1153delTTG | c.1379_1380insC |  |
|  |  |  |  | c.1385dupG |  |
